# Audio computer-assisted self-interviewing (ACASI) may avert socially desirable responses about infant feeding in the context of HIV

**DOI:** 10.1186/1472-6947-5-24

**Published:** 2005-08-02

**Authors:** Anthony K Waruru, Ruth Nduati, Thorkild Tylleskär

**Affiliations:** 1 Centres for Disease Control and Prevention (CDC), Kenya Medical Research Institute (KEMRI), P.O Box 1578, 040100 Kisumu, Kenya; 2 Network for AIDS Researchers in Eastern and Southern Africa (NARESA), P.O Box 10654, 00100 Nairobi, Kenya; 3 Centre for International Health, University of Bergen, Armauer Hansen Bld, N-5021 Bergen, Norway

## Abstract

**Background:**

Understanding infant feeding practices in the context of HIV and factors that put mothers at risk of HIV infection is an important step towards prevention of mother to child transmission of HIV (PMTCT). Face-to-face (FTF) interviewing may not be a suitable way of ascertaining this information because respondents may report what is socially desirable. Audio computer-assisted self-interviewing (ACASI) is thought to increase privacy, reporting of sensitive issues and to eliminate socially desirable responses. We compared ACASI with FTF interviewing and explored its feasibility, usability, and acceptability in a PMTCT program in Kenya.

**Methods:**

A graphic user interface (GUI) was developed using Macromedia Authorware^® ^and questions and instructions recorded in local languages *Kikuyu *and *Kiswahili*. Eighty mothers enrolled in the PMTCT program were interviewed with each of the interviewing mode (ACASI and FTF) and responses obtained in FTF interviews and ACASI compared using *McNemar's *χ^2 ^for paired proportions. A paired *Student's t-test *was used to compare means of age, marital-time and parity when measuring interview mode effect and two-sample *Student's t-test *to compare means for samples stratified by education level – determined during the exit interview. A *Chi-Square *(χ^2^*test*) was used to compare ability to use ACASI by education level.

**Results:**

Mean ages for intended time for breastfeeding as reported by ACASI were 11 months by ACASI and 19 months by FTF interviewing (p < 0.001). Introduction of complementary foods at ≤3 months was reported more frequently by respondents in ACASI compared to FTF interviews for 7 of 13 complementary food items commonly utilized in the study area (p < 0.05). More respondents reported use of unsuitable utensils for infant feeding in ACASI than in FTF interviewing (p = 0.001). In other sensitive questions, 7% more respondents reported unstable relationships with ACASI than when interviewed FTF (p = 0.039). Regardless of education level, respondents used ACASI similarly and majority (65%) preferred it to FTF interviewing mainly due to enhanced usability and privacy. Most respondents (79%) preferred ACASI to FTF for future interviewing.

**Conclusion:**

ACASI seems to improve quality of information by increasing response to sensitive questions, decreasing socially desirable responses, and by preventing null responses and was suitable for collecting data in a setting where formal education is low.

## Background

Research conducted in low-income countries often yield contradictory results where sensitive issues are concerned even in similar studies and populations [1]. One source of bias is socially desirable responses in face-to-face (FTF) interviewing. Consequently, data from studies on stigma-associated diseases such as HIV/AIDS may be flawed with potentially far-reaching implications on decision-making and public health policies.

The foregoing argument implies that traditional FTF interviewing is unsuitable for collecting potentially sensitive data and calls for alternative methods of interviewing that increase privacy and are less confrontational. ACASI is a method of interviewing conducted in a private setting and the interaction happens only between the respondent and the computer. This person-machine interaction eliminates the need for respondents to reply in a socially desirable manner, as would be the case in FTF interviewing. Studies have shown that privacy provided by ACASI increases reporting of sensitive behaviours [2-7]. Apparently, this increased reporting is not attributable to random errors [4,8]. A plausible explanation given by Cooley and Turner suggests that increased reporting is because respondents are more willing to respond in an ACASI than in FTF interviewing [9]. ACASI is more so apt where education is low since it eases the response task [2,7,8,10]. ACASI program has other advantages such as preventing non-response by guiding respondents through the interview by providing prompts. Skips and branching are automated preventing punching errors occurring in manual data entry. With ACASI, the interview is standardised hence avoiding interviewer variations that would occur in many research settings where FTF interviewing is carried out with the assistance of several interviewers.

ACASI has been shown to be feasible among women in Africa [8]. However, information on its use for interviewing mothers with low education and on sensitive issues in a clinical setting was lacking.

## Methods

### Respondents

This study was carried within the PMTCT pilot project at the Karatina District Hospital, Nyeri, Kenya. Mothers enrolled in the project regularly visiting the antenatal clinic during a three-months period (Sept – Nov 2002) were invited to participate in the study. By the end of the study, we had approached 104 mothers and 80 of them had consented to be interviewed. This number formed the sample. *Kikuyu *and *Kiswahili *were the languages of instructions and questioning and all respondents were able to hear and understand either.

### Study design

Using a crossover design, all the respondents were interviewed with each method of interviewing (ACASI and FTF). The first block of 70 respondents were interviewed using ACASI first, and the second block of 10 respondents were interviewed using FTF first. In the subsequent interviews, respondents undertook the opposite interview from the one that they had the first time. To investigate preferences, and attitudes for the two interview modes, we asked respondents questions about their experiences with both methods of interviewing in an exit FTF interview.

### Study expectations and questions

In the context of HIV, mothers face stigma when choosing feeding options and they would want to conform to what is socially desirable. It was hypothesised that in FTF interview, respondents could give socially desirable responses. It was also hypothesised that ACASI could increase response rates for sensitive questions on relationships. The study sought to assess response differences when ACASI was used compared FTF interviewing. We also wanted to explore feasibility, usability, and acceptability of ACASI in a clinical setting.

### Study instruments and data analysis

To handle the questioning and provide instructions and cues to respondents, a graphic user interface (GUI) of the ACASI program was developed using Macromedia Authorware^® ^[11]. We adopted 52 questions (a part of the English version of the questionnaire used in the PMTCT project), which were translated into Kikuyu and Kiswahili versions. Audio recording for both the Kikuyu and Kiswahili questions was done using Audiotools^® ^shareware version [12]. Audio editing was done in Adobe Premiere^® ^[13]. Finally, individual audio clips were inserted to correspond to questions in the ACASI program and a test-run of the program.

**Figure 1:**
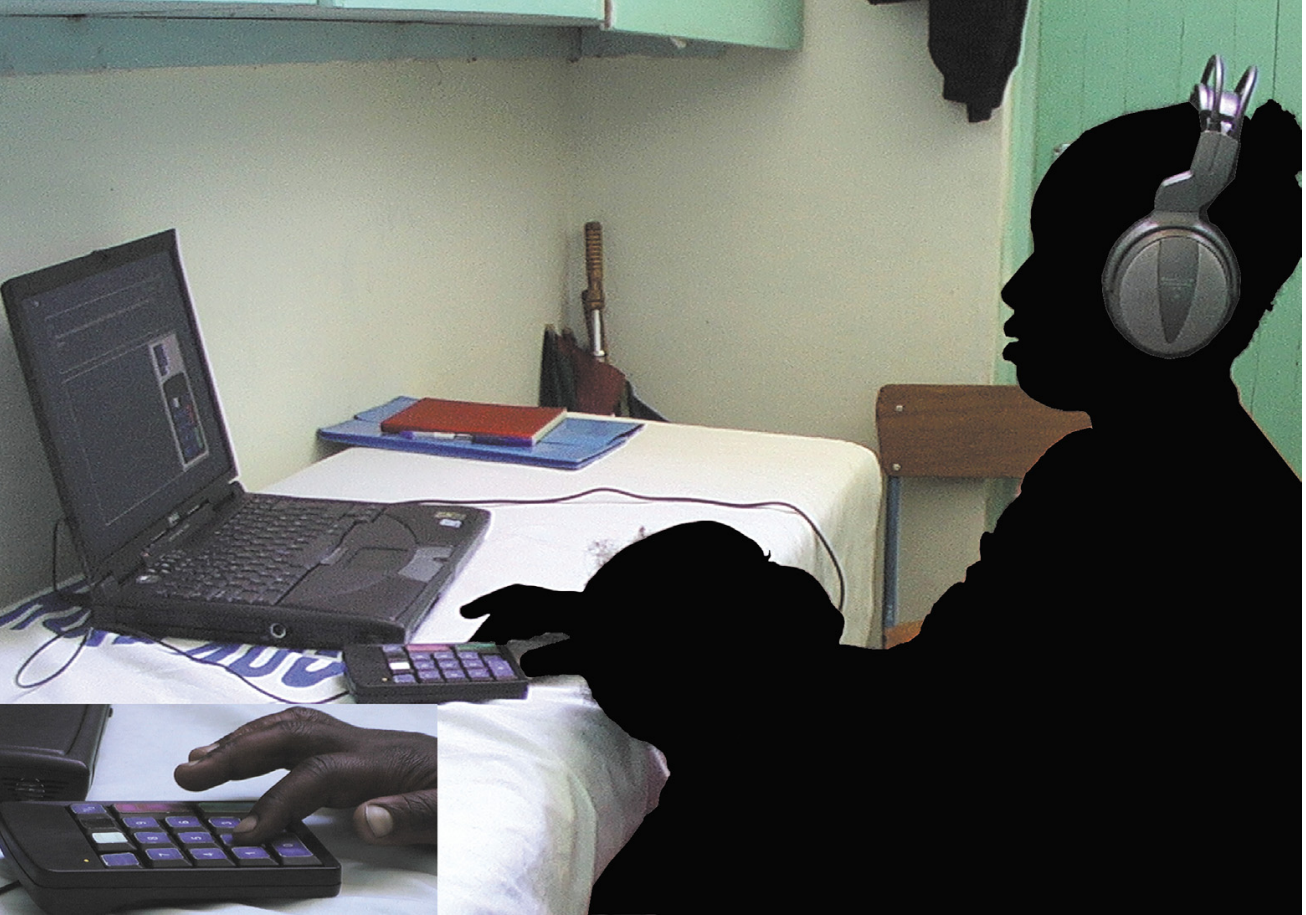
The audio computer-assisted self-interviewing (ACASI) set-up as it was used in Kenya. The mother being interviewed is sitting in front of a portable computer listening to the questions in her own language in the headset. She provides the answers by the small keypad with colour-coded buttons (enlarged bottom left). **Note**: Figure has been obscured for anonymity.

For implementation, a laptop, attached keypad (Targus™ Numeric keypad), and headphones were used (figure 1). All respondents went through two practice questions after which they were left on their own. It was necessary to guide respondents through the two practice questions in order to familiarise them with the use of the input device (external keypad) especially on how much pressure to exert on the keys since too much pressure would have resulted in repeated character entry. At the end of the ACASI, each interviewee's inputs were automatically added to a tab-delimited text record file and the amount of time that each respondent spent answering questions with ACASI was similarly recorded automatically. In the FTF interviewing inputs were directly entered by use of the keypad. At the end of the FTF interview, a differently named text file similar to the one produced in the ACASI was automatically generated. To determine preferences and attitudes for the two interviewing methods, we asked respondents questions about their experiences with both methods in an exit interview.

Data were analysed data using SPSS version 10.1 [14] and Epi 6.04 [15]. To compare means for age, marital-time and parity, we used a paired *Student's t-test *when measuring interview mode effect. We measured time taken by each mother to complete ACASI and used a *Student's t-test *to compare mean time taken by two groups of mothers – those educated up primary school level compared to mothers educated up to secondary school level. Level of education was determined for each mother from the exit interview. A *Wilcoxon Signed Rank *test was used for continuous variables for which distributions were skewed. For categorical variables, a *Pearson Chi-Square *(χ^2^*test*) was used to compare proportions for independent variables and when the expected value was less than 5, the *Fisher's Exact Test *was used. *McNemar's *χ^2^*test *was used to compare differences between paired proportions for categorical variables. For qualitative data, responses were reported literally and numbers of respondents giving similar responses indicated. Since the sample was larger than 30, we assumed normality in all analysis and a *p-value of < 0.05 *was considered statistically significant.

The Kenya PMTCT pilot project and the questionnaires used in this study had ethical approval from the Kenyatta national hospital ethical review committee and the national council of science and technology. The regional committee for medical research ethics, Norway-West also approved the study. Additional approval was not sought but all respondents orally consented to participate in the study.

## Results

### Demographic characteristics

Respondents comprised mostly of young mothers and their mean age was 25 years, (SD ± 5, range 17–38 years). Most respondents 49 (61%) had attained primary education and the rest 31 (39%) had attained secondary education. Most respondents (84%) were living in monogamous relationships. Mean duration for marital relationship was 4 years, (SD ± 4) by both modes of interviewing and parity ranged from 1 to 9. For all the demographic variables, data were not significantly different by mode of interviewing. Children to whom questions on infant feeding refer were more than 9 months old at the time of the interview.

### Infant feeding practices

On the intended time for breastfeeding, data varied significantly by mode of interviewing. Fourteen respondents (18%) indicated that they wanted to breastfeed their infants for an extended period of 24 months by ACASI while in the FTF interview, 44 (55%) indicated that they wanted to breastfeed their infants for 24 months (p < 0.001).

**Figure 2:**
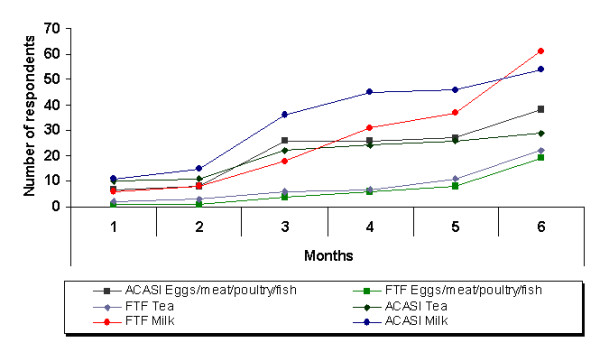
Three examples of difference in reporting infant feeding by method of interviewing: Audio computer-assisted self-interviewing (ACASI) or face-to-face (FTF). Number of mothers, who report giving milk or protein-rich foods (egg, meat or fish), whole milk and herbal tea to their babies according to the age of the child (x-axis). A higher proportion of mothers reported having given milk or protein-rich foods to their children below 6 months of age with the ACASI compared to FTF, interpreted as a reflection of the fact that it is socially desirable not to give milk or protein-rich foods before 6 months of age.

Respondents were asked whether they had introduced a selection of 13 food items (table 1). In all cases apart from 'baby formula' and 'fresh packet milk', the number of respondents who indicated that they had introduced the foods ≤3 months of age was fewer in the FTF interview than in the ACASI. These differences were significant (p < 0.05) for 7 food items compared to differences for only 3 food items in proportions of mothers who had introduced food items at 1 year of an infants age (table 1). A graphical presentation of 3 of the 13 food items: milk, protein-rich foods, and herbal tea is presented in (figure 2).

**Table 1:** Comparison of proportions of mothers who claimed to have introduced different food items at 3 and at 12 months by mode of interviewing: Audio computer-assisted self-interviewing (ACASI) or face-to-face (FTF) interview.

**Foods and fluids**	**At ≤ 3 months of age**			**At 1 year of age**		
	**ACASI n (%)**	**FTF n (%)**	**p ^a)^**	**ACASI n (%)**	**FTF n (%)**	**p ^a)^**

Plain water	54 (68)	50 (63)	ns ^b)^	72 (90)	79 (98)	0.016
Glucose water	30 (38)	25 (31)	ns	39 (49)	41 (51)	ns
Juice	12 (15)	3 (4)	0.012	18 (23)	12 (15)	ns
Tea	20 (25)	6 (8)	0.001	40 (50)	41 (51)	ns
Baby formula	4 (5)	4 (5)	ns	5 (6)	6 (8)	ns
Powder milk	0 (0)	0 (0)	ns	0 (0)	0 (0)	ns
Fresh packet milk	3 (4)	3 (4)	ns	6 (8)	5 (6)	ns
Cow's or goat milk	35 (44)	18 (23)	0.002	70 (88)	76 (95)	ns
Other fluids	27 (34)	23 (29)	ns	56 (70)	72 (90)	0.001
Fruits and vegetables	41 (51)	17 (21)	0.000	80 (100)	80 (100)	ns
Cassava and plantains	61 (76)	76 (95)	0.000	61 (76)	76 (95)	0.000
Starchy foods	31 (39)	17 (21)	0.016	69 (86)	72 (90)	ns
Eggs/poultry/fish/meat	26 (33)	4 (5)	0.000	56 (70)	56 (70)	ns

^a) ^McNemars χ^2 ^test

^b) ^ns not significant

Irrespective of interview mode, equal proportions of respondents 42 (53%) said they used a cup and spoon to feed their children in both ACASI and FTF interviewing respectively (table 2). However, more respondents reported use of a cup with holes on the snout in the ACASI than in the FTF interview (p = 0.001).

**Table 2:** Reported utensils used to feed infants by mode of interviewing: Audio computer-assisted self-interviewing (ACASI) or face-to-face (FTF) interview.

**Utensils**	**ACASI**	**FTF**
Cup	15 (19%)	6 (8%)
Cup and spoon	42 (53%)	42 (53%)
Closed cup with holes on the snout	20 (25%)	5 (6%)
Other	3 (4%)	27 (34%)
p ^a)^	0.001	

^a) ^McNemars χ^2 ^test

### Respondents' experiences with the ACASI program

We recorded time taken to complete the ACASI interview for 74 respondents. The mean time taken by mothers who had attained primary education was 25 minutes and 46 seconds and for mothers who had attained secondary education 25 minutes and 56 seconds a non-significant difference of only 10 seconds. In the exit interview, 56, (70%) of the respondents said they were able to answer questions without any problems and 24, (30%) indicated they had some hitches. However, of the 24, only 11 asked for help and the rest were able to continue on their own. Sixteen, (20%) of the respondents did not press the repeat key. Of the 64 who did, 61 pressed only a few times -usually once- while the other three pressed the button often. All the respondents listened to the recorded audio questions and instructions at all times. Ability to use ACASI was not different across education levels (table 3).

**Table 3:** Comparison of the ability to use audio computer-assisted self-interviewing (ACASI) between mothers with primary versus secondary education.

**Indicators for ability to use ACASI**	**Primary education (n = 49)**		**Secondary education (n = 31)**		**p ^a)^**
	
	**Yes**	**No**	**Yes**	**No**	
Difficult questions	4	45	5	26	0.298 ^b)^
Unable to answer a question	16	33	8	23	0.515
Asked for help	9	7	2	6	0.303 ^c)^
Pressed repeat key	42	7	22	9	0.108

^a) ^Pearson χ^2^

^b) ^Fisher's exact test used

^c) ^The question was relevant for only those who were unable to answer a question

### Preferences for and attitudes to mode of interviewing

Most respondents 52 (65%) preferred ACASI to FTF interviewing and the rest preferred either FTF (18 respondents, 23%), or both methods of interviewing (10 respondents, 13%). Preference though, did not vary by education level. Major reasons given for ACASI preference were: privacy, faster interviewing, and aspects of program usability such as (audio repetitions and prompts, the simultaneous on-screen text, clear instructions, the easy-to-use colour-coded number keypad). Some preferred FTF interviewing because the interviewer could clarify questions and respondents could ask other questions related to their own health in a FTF encounter. Three respondents felt that the computer was rigid in the way it asked questions.

### Perceived privacy and confidentiality

Majority of the respondents (84%) thought ACASI offered more privacy than FTF interviewing. Five respondents thought FTF interviewing offered more privacy, 3 considered both methods equally private and 5 considered neither method private. Preference and perceived privacy were similar between both levels of education.

To assess the importance that respondents assigned to confidentiality, we asked them to tell us how important it was for their responses not to be seen by others. Twenty-six, (33%) indicated that '*it did not matter*' while the majority 54 (68%) indicated that '*it was important that others do not see their responses'*.

## Discussion

This is the first study comparing ACASI and FTF conducted in an African rural clinical setting among mothers with low formal education. ACASI and FTF interviewing yielded similar results to neutral questions but differed consistently whenever questions of a sensitive nature were asked. Since our aim was not to validate any of the methods of interviewing, any of the two methods could have yielded the more correct answers. However, consistency of data divergence between the methods suggests that ACASI yielded responses that were less influenced by perceptions of what was socially desirable compared to FTF interviewing.

At the antenatal clinic, the nurses stress in their morning health talk sessions the importance of exclusive breastfeeding for the first 6 months of an infant's life and advocate for extended breastfeeding for at least two years. Our study demonstrates early (≤ 3 months) and extensive introduction of food items, irrespective of interview mode. This information concurs with a study done in Malawi showing that exclusive breastfeeding is uncommon and complementary foods are introduced early in rural families [16]. In the ACASI mode of interviewing even higher proportions of mothers reported early introduction of feeds, which suggests that the FTF responses were biased towards socially desirable responses.

Our data also indicates a bias of the FTF data in the direction of socially desirable responses for the intended length of breastfeeding. A larger proportion of mothers responded that they intended to breastfeed their infants for 24 months during FTF interviewing (44 respondents, 55%) as opposed to during ACASI (11 respondents, 14%). A plausible reason would be that respondents would want to confine their responses in FTF interviews to what they had been taught during the health talk sessions. Reported mean age of introducing protein-rich foods such as meat, eggs, or fish by ACASI mode was 117 days (approximately 4 months) and 173 days (approximately 6 months) by FTF interviewing. This discrepancy could mean that respondents knew that they should introduce protein-rich foods when their babies were older (as was taught during health talks) but in practice, they introduced protein-rich foods to babies at a young age.

More respondents (25%) indicated that they used a closed cup with holes on the snout for infant feeding in the ACASI interview compared to 6% in the FTF interview. Since this utensil is hard to clean, its use is discouraged in the morning health talks. It appears that the respondents gave socially desirable responses in the FTF interview hence the lower proportion reporting.

This study had some limitations. The ratio of respondents who undertook each of the interviewing methods in the first and subsequent interview were dissimilar (70:10 – ACASI first to FTF interviewing first). This was not due to design but because of lack of sufficient number of respondents in the second sequencing arm and we could not therefore effectively measure the effect of sequencing on responses. However, changing the order of interviewing only resulted to a decline in time respondents took to complete the ACASI interview among those who started on FTF interview first and ACASI second (*t (72) = 2.626 p = 0.011, CI: 2 – 13 minutes) *and had no effect on responses. Secondly, assignment of respondents to each of the interviewing sequencing arms was not random since it was done as a group of 70 (ACASI interviewing first) and the second group of 10 (FTF interviewing first) rather than an alternate assignment of respondents during recruitment to each of the interviewing mode.

## Conclusion

Studies have shown that ACASI increases reporting of sensitive issues in high-income countries but until recently, few studies had tried this methodology in resource-poor settings. This is the first study on ACASI conducted in an African rural clinical setting among mothers with low formal education. Our study results are in agreement with results of several studies that have shown that ACASI increases reporting of sensitive issues [2-7]. Differences in responses with the two methods of interviewing show that data on infant feeding in the sensitive context of HIV when collected during face-to-face interviews may be influenced by the respondents tendency to give socially desirable responses. Relying on such data may lead to imprecise decisions on public health issues. Use of ACASI in research or program settings involving sensitive issues can improve data, information and conciseness of decision-making.

## Competing interests

The author(s) declare that they have no competing interests.

## Authors' contributions

A Waruru participated in all aspects of the study including planning, design of the ACASI program, data collection and data analysis, and in writing the manuscript, R Nduati designed the initial questionnaire, prepared the study site, supervised data collection, participated in analysis and interpretation of data and in the writing of the manuscript. T Tylleskär had the initial idea for the study, supervised the study planning, the design of the ACASI interviews and participated in the analysis and interpretation of data and in the writing of the manuscript.

## Pre-publication history

The pre-publication history for this paper can be accessed here:


